# Computed tomography based analysis of the lamina papyracea variations and morphology of the orbit concerning endoscopic surgical approaches^☆^

**DOI:** 10.1016/j.bjorl.2018.04.008

**Published:** 2018-05-18

**Authors:** Gülay Açar, Mustafa Büyükmumcu, İbrahim Güler

**Affiliations:** a Necmettin Erbakan University, Meram Faculty of Medicine, Department of Anatomy, Konya, Turkey; b Selcuk University, Faculty of Medicine, Department of Radiology, Konya, Turkey

**Keywords:** Computed tomography, Endoscopic approach, Lamina papyracea, Orbital morphometry, Reconstructive surgery, Tomografia computadorizada, Abordagem endoscópica, Lamina papirácea, Morfometria orbital, Cirurgia reconstrutora

## Abstract

**Introduction:**

Radiologic evaluation is mandatory to assess the type of endoscopic approach concerning sinonasal pathology and reconstruction of fractured defects before any treatment modalities are instituted related to medial wall of the orbit.

**Objective:**

The goal was to provide improved understanding of the lamina papyracea variations and the relationship with the orbital morphometry.

**Methods:**

This retrospective study was performed using computed tomography scans of 200 orbits and results were compared with respect to age, sex, laterality and LP variations.

**Results:**

Lamina papyracea variations were categorized as type A, 80.5% (161/200); type B, 16% (32/200); type C, 3.5% (7/200). For medial wall the anterior and posterior lamina papyracea heights and angles were found as 17.14 mm, 147.88° and 9.6 mm, 152.72°, respectively. Also, the length of the lamina papyracea, the mean area of the orbital floor, medial wall, lamina papyracea and orbital entrance were 33.3 mm, 7.2 cm^2^, 6.89 cm^2^, 4.51 cm^2^ and 12.46 cm^2^ respectively. The orbital height and width were measured as 35.9 mm and 39.2 mm respectively. The mean orbital cavity depth was 46.3 mm from optic foramen to the orbital entrance and the orbital volume was 19.29 cm^3^. We analyzed the morphometric measurements tending to increase with aging and greater in men and the relationship of them with lamina papyracea types.

**Conclusion:**

Precise knowledge of the lamina papyracea anatomy using computed tomography is essential for safer and more effective surgery and preforming the dimensions of an implant. In this way, the postoperative complications can be decreased and the best outcome can be provided.

## Resumo

**Introdução:**

A avaliação radiológica é mandatória para avaliar o tipo de abordagem endoscópica no tratamento cirúrgico de doença nasossinusal e na reconstrução de fraturas antes de quaisquer modalidades de tratamento relacionadas à parede medial orbital.

**Objetivo:**

O objetivo foi proporcionar uma melhor compreensão das variações da lâmina papirácea e a relação com a morfometria orbital.

**Método:**

Este estudo retrospectivo foi realizado por meio de tomografia computadorizada de 200 órbitas, e os resultados foram comparados em relação à idade, sexo, lateralidade e variações da lâmina pairácea.

**Resultados:**

As variações da lâmina papirácea foram categorizadas como tipo A, 80,5% (161/200); tipo B, 16% (32/200); tipo C, 3,5% (7/200). Para a parede medial, as medidas das alturas anteriores e posteriores da lâmina papirácea e ângulos foram de 17,14 mm, 147,88° e 9,6 mm, 152,72°, respectivamente. Além disso, as medidas do seu comprimento da, da área média do assoalho orbital, e da parede medial, lâmina papyracea e entrada orbital foram: 33,3 mm, 7,2 cm^2^, 6,89 cm^2^, 4,51 cm^2^ e 12,46 cm^2^, respectivamente. As medidas da altura e da largura orbitais foram 35,9 mm e 39,2 mm, respectivamente. A profundidade média da cavidade orbital foi de 46,3 mm, do forame óptico até a entrada orbital, e o volume orbital foi de 19,29 cm^3^. Analisamos as medidas morfométricas com tendência a aumentar com o envelhecimento e nos indivíduos do sexo masculino, e a relação das mesmas com os tipos de lâmina.

**Conclusões:**

O conhecimento preciso da anatomia da lâmina papirácea por meio de tomografia computadorizada é essencial para uma cirurgia mais segura e eficaz, além de permitir pré-moldar as dimensões do implante. Assim, as complicações pós-operatórias podem ser minimizadas, obtendo-se melhores resultados.

## Introduction

The lamina papyracea (LP) is the weakest point of the medial wall of the orbit, which forms a connecting line between paranasal sinuses and the orbit. Also, the ethmoidal foramina along the LP are life-saving anatomical landmarks and allow transverse passage of the ethmoidal arteries into ethmoidal cells.^1^ During surgical interventions such as reconstruction and endoscopic approaches, success in surgical strategy and planning mainly rely on the surgeon's detailed knowledge of the LP variations and the location of ethmoidal foramina, providing a shorter surgery time and avoidance of complications.^2^, ^3^, ^4^

During endoscopic sinus surgery (ESS) which is essential for the treatment of chronic rhinosinusitis and removal of the sinonasal pathologies, accidental LP injury can be incurred.^4^, ^5^ Precise knowledge of LP localization according to inferior nasal turbinate (INT) attachment to the lateral nasal wall is essential to avoid orbital penetration.^4^ As a consequence of LP penetration, periorbital ecchymosis or emphysema, venous orbital hematoma, medial rectus injury, and blindness can occur. Also, revision ESS can be required for residual unopened ethmoid cells at the LP due to insufficient knowledge of the radiological and endoscopic sinonasal anatomy.^4^, ^6^

On the other hand, reconstruction is indicated in cases of visual acuity, diplopia with extraocular muscle entrapment, large bony defects, enophthalmos and cosmetic complications.^7^, ^8^ The inferomedial strut (IMS), the anterior (AEF) and posterior ethmoidal (PEF) foramina identified on CT coronal plane are important surgical landmarks for a combined repair of orbital bony defects.^1^, ^9^ The combined orbital fractures involving the IMS represent an application difficulty of implant material in reconstruction surgery. In addition, an adequate surgical exposure and access as well as an implant with proper size and shape is essential to insure the success of surgical technique.^10^, ^11^ Also, the lack of malleability of thick implants, the close proximity of the implant to vital orbital structures, and incorrect estimation of the dimensions of the implant can cause significant postoperative complications such as enophthalmos, optic neuropathy secondary to implant impingement, orbital emphysema and hemorrhage.^7^, ^10^, ^11^

By using multiplanar reconstruction (MPR) detailed knowledge of the normal anatomy of the bony orbit and relationship with the surrounding structures can be acquired.^12^, ^13^ Radiologic description of the size and morphology of the medial wall of the orbit on CT scan is essential to guide the surgeon in diagnosis, deciding the proper surgical approach and outcome prediction.^13^

In this study, we focused on the LP variations and orbital medial wall morphometry from the point of view of endoscopic sinus surgery and reconstruction of fractures. To understand the medial wall of the orbit precisely, we measured a set of angular and linear parameters by using facial MPR images. In particular, we analyzed the relationship of these morphometric parameters with LP variations. The CT scan analysis of these parameters may be helpful to the surgeon in preoperative planning and avoiding accidental penetration of the LP.

## Methods

This retrospective study was approved by our local Ethics Committee with an approval number 2016/539 and performed using facial (orbital and paranasal) CT images of 100 patients, ranging from 18 to 90 years of age, who presented to the Department of Radiology for clinical purposes between September 2015 and July 2016. The patients who had orbital fractures, paranasal sinuses with any distorting pathologies which damaged the orbital bony contours, congenital deformities and Graves orbitopathy were excluded.

All patients were evaluated using 128 slice CT scanner (Siemens, imaging parameters: Kv = 120; mA = 160; rotation time, 0.5 s; collimation, 128 × 0.625; FOV = 220 mm). MPR images (associated coronal and sagittal images of 1 mm slice thickness) were generated on the basis of the axial images which were obtained with a section thickening of 0.625 mm. According to a predesigned protocol on Syngo Via (Siemens, Germany) the CT images were reconstructed and analyzed by the two investigators (a radiologist and an anatomist). Also, all results were checked for accuracy by them. The patients consisted of 23 females (43%) and 77 males (57%) with a median age of 48.60 ± 12.32 years for females and 37.36 ± 15.24 years for males. We divided the patients into 3 age groups: Group 1 (18–39 y) including 94 orbits, Group 2 (40–59 y) including 78 orbits; Group 3 (60–90 y) including 28 orbits. We described the anatomic landmarks using measurements in Table 1.

**Table 1:** Definitions of measurements of the orbital morphometry.

Definitions	
Orbital entrance height	The distance from the infraorbital canal to superior orbital rim
Orbital entrance width	The distance from the frontoethmoidal suture to midpoint of the lateral rim
Orbital cavity depth	The distance from the orbital foramen to the plane passing across the orbital entrance
Orbital entrance area	The area of an octagonal polygon which is formed by anatomical landmarks; supraorbital foramen, frontoethmoidal suture, infraorbital foramen, zygomaticofrontal suture and midpoints between them
Orbital floor area	The triangular area from the orbital foramen to the plane passing across the orbital entrance
Orbital medial wall area	The triangular area from the orbital foramen to the plane passing across the orbital entrance
Anterior orbital medial wall height and inferiomedial angle	The distance from the AEF to the ethmoidomaxillary suture and inferomedial angle between the orbital medial and inferior walls
Posterior orbital medial wall height and inferiomedial angle	The distance from the AEF to the ethmoidomaxillary suture and inferomedial angle between the orbital medial and inferior walls
Lamina papyracea length	The distance from the posterior lacrimal crest to junction point between sphenoid and ethmoid sinuses

**Figure 1:**
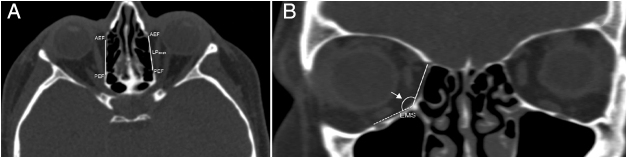
(A) Axial image identifying the location of the anterior (AEF) and posterior ethmoidal foramen (PEF) shows the measurement of lamina papyracea length (LP_length_); (B) coronal image shows the anterior lamina papyracea height (LP_height_) from the anterior ethmoidal foramen to the ethmoidomaxillary suture (EMS) and the anterior inferomedial angle (arrow) between medial and inferior walls the orbit.

**Figure 2:**
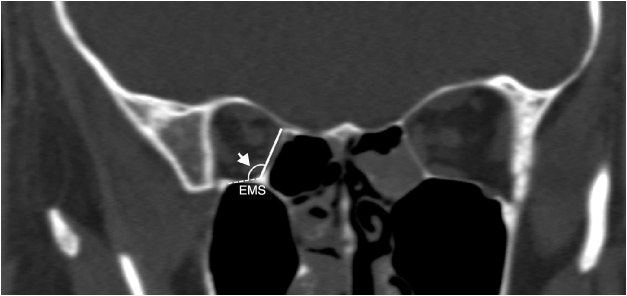
Coronal image shows the posterior lamina papyracea height (LP_height_) from the posterior ethmoidal foramen to the ethmoidomaxillary suture (EMS) and the posterior inferomedial angle (arrow) between medial and inferior walls the orbit.

**Figure 3:**
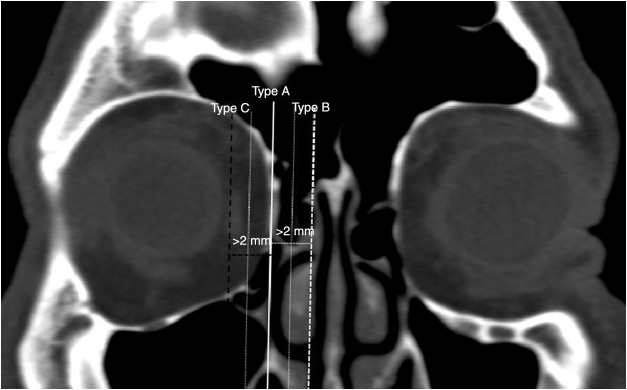
Coronal image shows the lamina papyracea variations related with inferior nasal turbinate attachment (white vertical axis) to the lateral nasal wall. Type A (white thin dotted axis); located within 2 mm on either side of the vertical axis; Type B (white thick dotted axis); medial to axis by >2 mm; Type C (black thick dotted axis); lateral to axis by >2 mm.

We determined the AEF and PEF, and measured the length of the LP between them in an axial plane (Fig. 1A). Then, in coronal plane we measured the anterior and posterior LP heights as a distance from the AEF and PEF to the IMS, which was the junction between the orbital medial wall and floor. Also, the anterior and posterior inferomedial angles at the IMS were measured (Figure 1, Figure 2). Conversely, we accepted the LP as a trapezoid and calculated the area of the LP by using the formula; LP length/2 × (anterior LP height + posterior LP height). Also, we analyzed the types of the LP position related with INT attachment to the lateral nasal wall (Fig. 3). The vertical axis was located on the INT attachment in the coronal plane. We categorized the LP into three types as follows, which were based on Herzallah et al.’s^4^ classification of the LP type:

Type A, located within 2 mm on either side of the vertical axis;
Type B, medial to axis by >2 mm;
Type C, lateral to axis by >2 mm.

**Figure 4:**
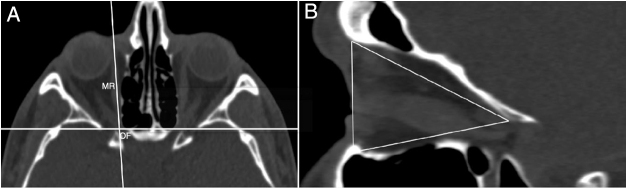
(A) Axial image shows identification of the orbital foramen (OF) and medial rectus muscle (MR). (B) The measurement of the area of the medial wall in sagittal plane of axial position.

**Figure 5:**
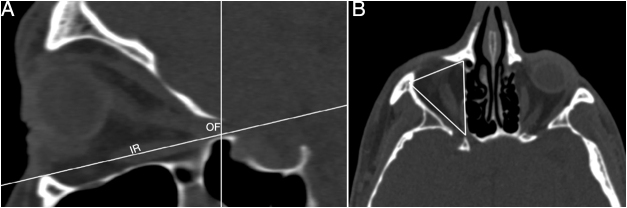
(A) Sagittal image shows identification of the orbital foramen (OF) and inferior rectus muscle (IR). (B) The measurement of the area of orbital floor in axial plane of sagittal position.

The intersection of ‘*x*’ and ‘*y*’ axes was located at the orbital foramen (OF) and the vertical axis passed along the medial rectus muscle in the axial plane (Fig. 4A). We measured the area of the medial wall at this position in sagittal plane (Fig. 4B). Then, the intersection of the axes was located at the OF and the horizontal axis passed along inferior rectus muscle in the sagittal plane (Fig. 5A). We measured the area of the inferior wall at this position in the axial plane (Fig. 5B).

**Figure 6:**
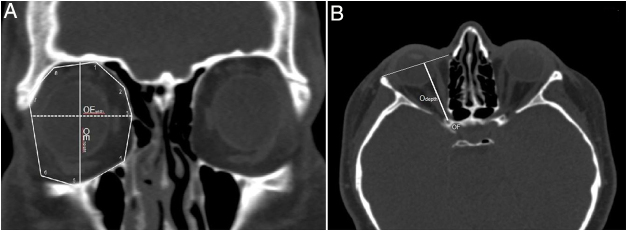
Multiplanar reconstruction of the CT images showing the morphometric measurements regarding orbital entrance and orbital depth. (A) The orbital rim landmarks were identified on octagonal polygon shaped orbital entrance in coronal plane: 1 supraorbital foramen, 3 frontoethmoidal suture and 2 midpoint between them, 4 ethmoidomaxillary suture, 5 infraorbital foramen, 7 zygomaticofrontal suture and 6 midpoint between them, 8 midpoint between zygomaticofrontal suture and supraorbital foramen. The area of this octagonal polygon was measured as orbital entrance area. Orbital entrance height (OE_height_) from infraorbital foramen to midpoint of the superior orbital rim and orbital entrance width (OE_width_) from frontoethmoidal suture to midpoint of the lateral orbital rim. (B) Orbital depth was taken as horizontal measurement from orbital foramen (OF) to orbital entrance.

In addition, we aimed to describe the morphometry and geometry of the orbit from the point of view of an endoscopic reconstructive surgery and to provide a detailed knowledge about the size and shape of the implant. We formed an octagonal polygon on orbital rims by specifying 8 anatomic landmarks and measured the area of the orbital entrance (OE). These orbital rim landmarks, which were visually identifiable and similar to the morphometric parameters in previous studies included; supraorbital foramen superiorly, the frontoethmoidal suture medially, infraorbital foramen inferiorly, the zygomaticofrontal suture laterally and midpoints between them (Fig. 6A). Also, the vertical measurement between the infraorbital canal and superior orbital rim, and the horizontal measurement from the frontoethmoidal suture to midpoint of the lateral rim were taken as an OE height and width, respectively (Fig. 6A). On the other hand, the optic nerve was viewed as uninterrupted in the axial plane and the depth of the orbital cavity from the OF to the plane passing across the OE was measured (Fig. 6B). We assumed the orbit as an octagonal pyramid-shaped bony compartment and calculated the orbital volume by using the formula; 1/3 × OE area × orbital depth.

On the other hand, we compared the measurement values with respect to age, sex, laterality and LP types. SPSS 22 (SPSS, Inc., Chicago, IL, USA) was used for statistical analysis. For statistical comparisons, Chi-square test, *t*-test, One-Way Analysis of Variance (ANOVA) were used. The significance level was set at *p* < 0.05.

## Results

We measured the mean length of the LP as 33.3 ± 2.9 mm and calculated the mean area of the trapezoid LP as 4.51 ± 0.81 cm^2^. The mean lengths of the anterior and posterior LP height at the level of the AEF and PEF in the coronal scan were found as 17.4 ± 2.7 mm and 9.6 ± 2.5 mm, respectively. Also, the mean inferomedial angular measurements including AEF and PEF levels were 147.88° ± 7.54° and 152.72° ± 9.61°, respectively. We measured the mean values of the medial and inferior wall areas as 7.20 ± 0.6 cm^2^ and 6.89 ± 0.59 cm^2^, respectively. Both the mean OE height, width and area measurement values were 35.9 ± 1.7 mm, 39.2 ± 2.0 mm and 12.46 ± 0.70 cm^2^, respectively. We reported the mean values of orbital depth and volume as 46.3 ± 2.2 mm and 19.29 ± 1.61 cm^3^, respectively. All morphometric measurement values showed significant differences with respect to sex and age as seen in Table 2, Table 3, although there was no statistically significant difference among right and left sides. In Table 2, we demonstrated that all measurement values were higher in males than females and showed statistically a significant difference except for the angular results. The measurement results indicated that subjects in the first and second age groups showed statistically significant differences in orbital height, width and depth, medial and inferior wall areas and anterior LP height as shown in Table 3.

**Table 2:** The distribution of the comparison in morphometric measurements between females and males.

Morphometric measurements	Female	Male	Total
	Mean ± SD	Mean ± SD	Mean ± SD
*Orbital entrance height (mm)*	35.0 ± 1.3^b^	36.2 ± 1.8^b^	35.9 ± 1.7
*Orbital entrance width (mm)*	38.5 ± 1.6^a^	39.4 ± 2.1^a^	39.2 ± 2.0
*Orbital entrance area (mm*^*2*^*)*	1205 ± 66^b^	1258 ± 67^b^	1246 ± 70
*Orbital cavity depth(mm)*	45.5 ± 2.4^a^	46.5 ± 2.0^a^	46.3 ± 2.2
*Orbital volume (mm*^*3*^*)*	1878 ± 167^b^	1997 ± 150^b^	1929 ± 161
*Orbital floor area (mm*^*2*^*)*	659 ± 57^b^	698 ± 57^b^	689 ± 59
*Orbital medial wall area (mm*^*2*^*)*	688 ± 56^b^	730 ± 59^b^	720 ± 60

*Orbital medial wall height (mm)*			
Anterior	16.2 ± 3.4^a^	17.6 ± 2.4^a^	17.4 ± 2.7
Posterior	9.0 ± 2.5^a^	9.8 ± 2.4^a^	9.6 ± 2.5

*Orbital inferiomedial angle (°)*			
Anterior	147.82 ± 8.45	147.89 ± 7.28	147.88 ± 7.54
Posterior	151.92 ± 8.11	152.96 ± 10.02	152.72 ± 9.61

*Lamina papyracea length (mm)*	32.5 ± 2.9^a^	33.5 ± 2.9^a^	33.3 ± 2.9
*Lamina papyracea area (mm*^*2*^*)*	418 ± 75^a^	460 ± 81^a^	451 ± 81

Mean ± SD (standard deviation).

Unpaired *t*-test.

a *p* < 0.05.

b *p* < 0.001.

**Table 3:** Comparisons of the morphometric measurements among the three age groups.

Morphometric measurements	Total	18–39 y	40–59 y	60–90 y
Orbital entrance height (mm)	35.9 ± 1.7	35.3 ± 3.0^a^	36.4 ± 2.1^a^	36.1 ± 0.6
Orbital entrance width (mm)	39.2 ± 2.0	38.8 ± 2.3^a^	39.4 ± 0.6^a^	39.0 ± 0.4
Orbital entrance area (mm^2^)	1246 ± 70	1185 ± 11	1266 ± 11	1254 ± 20
Orbital cavity depth (mm)	46.3 ± 2.2	45.7 ± 1.2^a^	46.4 ± 1.0^a^	46.2 ± 0.3
Orbital volume (mm^3^)	1929 ± 161	1851 ± 13	1957 ± 14	1933 ± 20
Orbital floor area (mm^2^)	689 ± 59	673 ± 10^a^	699 ± 46^a^	686 ± 64
Orbital medial wall area (mm^2^)	720 ± 60	689 ± 39^a^	726 ± 17^a^	710 ± 48
Anterior	17.4 ± 2.7	16.4 ± 1.0^a^	17.7 ± 1.2^a^	17.5 ± 1.5
Posterior	9.6 ± 2.5	9.5 ± 3.3	10.1 ± 0.3	9.8 ± 0.7
Anterior	147.88 ± 7.54	148.70 ± 14.6	147.39 ± 13.6	147.33 ± 21.0
Posterior	152.72 ± 9.61	152.88 ± 3.70	152.56 ± 10.3	152.45 ± 3.81
Lamina papyracea length (mm)	33.3 ± 2.9	33.7 ± 3.5	33.5 ± 3.5	33.0 ± 2.6
Lamina papyracea area (mm^2^)	451 ± 81	460 ± 32	455 ± 68	446 ± 10

ANOVA (One-Way Analysis of Variance).

a *p* < 0.05.

The distributions of 200 LP types were categorized as follows; Type A, 80.5% (161/200); Type B, 16% (32/200); Type C, 3.5% (7/200). There were no statistically significant differences in prevalences with respect to sex, age and laterality. In addition, we analyzed the relationship between morphometric measurements and LP types. As seen in Table 4, all mean values except the orbital depth and LP length were smallest in Type C, whereas the inferior wall area, anterior and posterior LP heights, anterior inferomedial angle and LP length values showed statistically significant differences between Type B and C.

**Table 4:** The relationship between the morphometric measurements and the lamina papyracea location types.

Morphometric measurements	Lamina Papyracea-inferior nasal turbinate attachment type		
	Type A	Type B	Type C
*Orbital entrance height (mm)*	36.0 ± 1.8^a^	35.7 ± 1.3	34.8 ± 2.4^a^
*Orbital entrance width (mm)*	39.5 ± 1.9^a^	39.2 ± 1.8	38.2 ± 4.3^a^
*Orbital entrance area (mm*^*2*^*)*	1267 ± 68	1244 ± 66	1225 ± 128
*Orbital cavity depth (mm)*	46.3 ± 2.1	45.9 ± 2.5	46.9 ± 1.9
*Orbital volume (mm*^*3*^*)*	1943 ± 154	1924 ± 177	1923 ± 257
*Orbital floor area (mm*^*2*^*)*	694 ± 55^a^	670 ± 73	646 ± 53^a^
*Orbital medial wall area (mm*^*2*^*)*	725 ± 61	710 ± 49	701 ± 71

*Orbital medial wall height (mm)*			
Anterior	17.4 ± 2.8	17.7 ± 2.2^a^	15.7 ± 1.7^a^
Posterior	9.6 ± 2.6	9.8 ± 1.9^a^	9.0 ± 2.1^a^

*Orbital inferior-medial angle (°)*			
Anterior	147.51 ± 7.55	150.87 ± 6.78^a^	142.73 ± 6.77^a^
Posterior	152.66 ± 10.11	152.71 ± 7.46	152.17 ± 6.75

*Lamina papyracea length (mm)*	33.4 ± 2.9	32.2 ± 2.8^a^	34.9 ± 3.4^a^
*Lamina papyracea area (mm*^*2*^*)*	453 ± 84	444 ± 73	431 ± 54

Type A, lamina papyracea located within ≤2 mm on either side of the inferior nasal turbinate attachment; Type B, medial to inferior nasal turbinate attachment by >2 mm; Type C, lateral to inferior nasal turbinate attachment by >2 mm.

Mean ± standard deviation.

ANOVA (One-Way Analysis of Variance).

a *p* < 0.05.

## Discussion

The medial wall of the orbit contributes significantly to maintain orbital volume and the displaced or combined fractures and perforation of the LP result in a risk of enophthalmos. The notches in the LP, which can be identified on coronal CT image are a good reference for the position and orientation of the ethmoidal arteries.^1^, ^10^, ^14^ For the removal of the pathologies in paranasal sinuses and reconstruction of normal orbital anatomy, CT imaging is the primary technique using to view LP variations or dehiscence and to localize the ethmoid air cells and hypoplastic maxillary sinus.^4^, ^8^, ^14^ Also, MPR allows observation of craniofacial bones from various angles and an evaluation of the sinonasal pathologies and complex fractures with overlapping fragments or minimal displacement.^15^, ^16^ Recognition of the CT appearance of the normal LP anatomy allows assessment of the goals of ESS and reconstructive surgery including meticulous planning, the use of adjunctive surgical approaches, and efficient clearance of ethmoid air cells on the LP, avoiding its injury and proper implant dimensions.^17^ In radiological anatomical studies of the anatomical landmarks on orbital walls, the area and volume of the orbital cavity were analyzed to guide a surgeon in the choice an appropriate endoscopic approach and to avoid surgical complications.^12^, ^14^, ^18^, ^19^

Many clinical problems including, nasal polyposis, hypoplastic maxillary sinus, uncinectomy, middle meatal antrostomy, severe epistaxis, orbital decompression and reconstruction may require an endoscopic surgical approach to the LP.^2^, ^4^ During ESS, an unexpected penetration of the LP, which is located just superior to the maxillary antrostomy, can cause orbital complications such as massive hemorrhage, orbital hematoma, blindness and optic neuropathy.^4^, ^5^, ^14^ Shigeta et al. reported the prevalence of the LP injury in their prospective study as 5.8%.^5^ There were different grades of nasal polyposis affecting up to 4% of the population, and also, larger polyps represent a risk factor during ESS.^6^ Also, Gore et al. demonstrated that residual ethmoid air cells on the LP were identified in 79% of revision ESS patients.^20^ So, radiologic identification of the LP positions from the perspective of endoscopic approach can be of great help in efficient clearance of ethmoid air cells on the LP and avoidance of penetration. Also, it can also provide a baseline for future studies to identify LP variations associated with higher risk of injury or residual disease.^4^, ^6^, ^14^

In the literature, the prevalence of the pure medial orbital wall fractures was higher than floor fractures and the ratio of them around 1.8:1.^11^, ^21^ Although the weakest point of the medial wall is the LP, the bony septa between the bullae of ethmoid pneumatization which appear honeycombed shape supports the LP against trauma forces. But, the combined orbital wall fractures mostly occur at IMS and cause the loss of internal bony support.^1^, ^11^ Also, reconstructive surgeons may be confronted with difficult patients such as those involving complex fractures: reconstruction of large defects presents challenges. The significant discrepancy between the implant shape and the normal anatomic shape of the orbital wall in fractures including IMS can result in insufficient orbital volume reduction.^7^, ^10^, ^21^ On the other hand, the AEF and PEF at the level of the frontoethmoid suture can serve as a vital anatomic reference to proper implant placement. As a result of inaccurate reduction and reconstruction of the orbital walls, residual complications such as enophthalmos, diplopia, retrobulbar hemorrhage from injury to the posterior ethmoidal artery and orbital emphysema can be seen.^7^, ^10^, ^11^ Therefore, revision surgery to treat them is required before excess scarring occurs. To avoid these complications it is necessary to be aware of the linear and angular orbital morphometry including the IMS.^7^ Because the medial wall of the orbit can be fractured easily and the LP is mostly used in endoscopic endonasal approach, knowledge of the anatomic dimensions of the medial wall increases the success rate of surgical technique and minimizes the iatrogenic complications.^22^

The AEF and PEF as a notch in the medial orbital wall were used to identify the dimensions of the LP. Also, these parameters can be used in confirmation of the posterior margin of the fracture, which is critical to prevent residual enophthalmos due to inadequate placement of the implant.^10^, ^11^, ^21^ Using CT scans Song et al. measured the anterior and posterior heights of the LP, the length of the LP and LP area as 15.32 mm, 11.04 mm, 30.50 mm and 4 cm^2^.^11^ Using 3D CT images Kang et al. reported the anterior and posterior heights of the LP and inferomedial angles as 17.73 mm, 12.76 mm, 132.118° and 136.88°, respectively.^21^ In our study, the mean values of the length, area, anterior and posterior heights of the LP and inferomedial angles were 33.3 ± 2.9 mm, 451 ± 81 cm^2^, 17.4 ± 2.7 mm, 9.6 ± 2.5 mm, 147.8° ± 7.54° and 152.72° ± 9.61°, respectively. All parameters showed a tendency to increase with aging and were greater in men, similar to the findings of Kang et al. Also, angular values did not show statistically significant differences with respect to sex, age, and laterality.

The CT scan analysis of surface area of the floor and medial orbital wall can offer accurate understanding of the size of the surgical window for resection or repair of the orbital and sinonasal pathology. In many previous studies, a baseline volume of normal bony orbit, which is useful in the prediction of enophthalmos, was measured and compared with volumetric changes in the fractured orbit.^12^, ^14^, ^19^ Felding et al. measured the volume and surface area of the orbital cavity as 24.27 ± 3.88 cm^3^ and 32.47 ± 2.96 cm^2^ by using unbiased stereological sampling technique.^12^ We calculated the mean volume of an octagonal pyramid-shaped bony orbit as 19.29 ± 1.61 cm^3^. Van Rompaey et al. measured the area of the orbital floor and medial wall of retrobulbar space as 4.33 cm^2^ and 3.34 cm^2^ by using CT scans.^14^ Using dry skulls, Fitzhugh et al. found the area of the orbital floor and medial wall as 7.14 cm^2^ and 6.58 cm^2^, respectively.^19^ We measured their mean values as 7.20 cm^2^ and 6.89 cm^2^ that were similar to Fitzhugh et al.’s. Also, these values were larger in males than females and showed a tendency to increase with aging as seen in Table 2, Table 3.

According to previous studies, there was a significant difference between the morphometric measurements of the orbital cavity which complicated to select the proper size of repairing materials.^7^, ^8^ The dimensions of octagonal shaped OE are an important factor for reconstructing the orbital rims and volume.^19^ Khademi et al., Ji et al. and Fitzhugh et al. found the orbital width and height as 32.14 mm, 33.35 mm, 33.9 mm and 28.49 mm, 39 mm, 38.7 mm, respectively.^18^, ^19^, ^23^ In our study, these values were found as 35.9 mm and 39.2 mm and larger in males than females, similar to the results in previous studies. Ji et al. measured the mean OE area as 11.80 cm^2^ similar to our value as 11.46 cm^2^.^23^ Khademi et al.^18^ found the orbital depth from OF to the OE as 38.84 mm, but we measured it as 46.3 mm. We reported that the OE measurement values were higher in male and increased significantly with age (*p* < 0.05), although there was no statistically significant difference with respect to laterality (Table 3).

During endoscopic sinus surgery, the lower limit of the middle meatal antrostomy was the inferior nasal turbinate attachment to the lateral nasal wall. If preoperatively the LP location related to this limit can be identified, the LP penetration, which was the most frequent iatrogenic complication (5%) was prevented.^4^ So, we classified the LP localization concerning endoscopic perspective and analyzed the relationship with the morphometric measurements in Table 4. Also, El-Anwar et al. described the positions of the LP in patients who had nasal polyp and reported the prevalence's of Type A, B, C as 60.6%, 18.6%, 21.3%.^6^ In our study, we demonstrated that the ratio of Type A, B, C in normal sinonasal anatomy was 80.5%, 16%, 3.5%. A medially located LP (Type B) that the least resistant to surgical interventions can be easily penetrated during uncinectomy. A laterally located LP (Type C) requires adequate clearance of ethmoid air cells which causes the LP penetration.^4^ El-Anwar et al. found that the patients who had larger polyps were associated with significantly more LP Type B than smaller polyp.^6^ In this study, the orbital depth and LP length were found as the longest in Type C and smallest in Type B. Both of the anterior and posterior medial wall heights and inferomedial angles were highest in Type B, smallest in Type C. The mean values of the inferior wall area, orbital height and width were significantly larger in Type A than Type C (*p* < 0.05). Also, the OE area, orbital volume and medial wall area showed a mild tendency to increase in Type A.

Compared with previous studies, our outcomes showed little diversity depending on the differences in reference landmarks, methodology and ethnicities. On the other hand, using MPR images we measured some parameters which were not used in previous studies such as an area of the OE which was resembled the octagonal polygon in shape, the anterior and posterior inferomedial angles, which were useful for reconstructive surgery. Also we analyzed the relationship between the LP variations and these measurement values concerning ESS. Thus, the comparative data was deficient. Nevertheless, the higher number of patients and future studies including measurements in children and patients with orbital fractures may give researchers more comprehensive results.

## Conclusion

In this study, we evaluated LP variations, the orbital morphometry and analyzed the relationship between them concerning endoscopic surgery in all respects. As a result, the LP variations can alter the orbital geometry and play a key role when choosing an appropriate surgical approach to avoid LP injury. These results again emphasized the value of preoperative CT imaging which can offer accurate understanding the regional anatomy of and around the bony orbit. The comprehensive knowledge of the normal anatomy of the LP allows safer and more effective sinus surgery and is essential for sufficient orbital reconstruction with proper implant, and also recreation of natural slope at the IMS. So, the success of surgical technique increases and the best outcome can be provided.

## Conflicts of interest

The authors declare no conflicts of interest.
